# Factors Influencing Long-Term Care Service Needs among the Elderly Based on the Latest Anderson Model: A Case Study from the Middle and Upper Reaches of the Yangtze River

**DOI:** 10.3390/healthcare7040157

**Published:** 2019-12-03

**Authors:** Liao Zeng, Xiaocang Xu, Chunxun Zhang, Linhong Chen

**Affiliations:** 1 Research Center for Economy of Upper Reaches of the Yangtse River, Chongqing Technology and Business University, Chongqing 400067, China; zengliao@ctbu.edu.cn; 2 School of Economics, Chongqing Technology and Business University, Chongqing 400067, China; 3 Department of Economics, Chongqing Technology and Business University, Chongqing 400067, China; tiantian20120424@126.com; 4 School of Mathematics and Statistics, Chongqing Technology and Business University, Chongqing 400067, China; 5 School of Public Administration, Sichuan University, Chengdu 610065, China

**Keywords:** long-term care, long-term care insurance, the elderly, the latest Anderson Model, psychosocial factors, income

## Abstract

The rapid growth of population aging makes providing adequate long-term care (LTC) services for the elderly a serious social dilemma in China. Thus, it is necessary to carry out a theoretical discussion on the LTC service needs of the elderly and find out their influencing factors. With four regions of the middle and upper reaches of the Yangtze River as the sample case, this study aims to explore the factors that affect LTC service needs of the elderly in the frame of the latest Anderson Model, which added psychosocial factors to predisposing characteristics, enabling factors, and need factors in the old version. Some interesting results have been found, for example, self-image evaluation is composed of several factors such as general physical health, attitude towards life, or psychosocial states. Finally, sub-analyses—namely, by age, by gender, and by educational level—were carried out since the choice of different long-term care service patterns is related to different age/gender/education groups.

## 1. Introduction

The aged tendency of population is becoming a major challenge for many countries including China. According to CHFPY (China health and family planning yearbook, 2017), people aged 65 or above was about 88 million (close to 7% of the total population) in 2000. However, in 2016, those people raised to 150.03 million, which is over 10.8% of the total population. The rapid growth of population aging makes providing adequate long-term care services for the elderly has becoming a serious social dilemma. For predicting the needs and costs of LTC (long-term care) services for the elderly, it is necessary to carry out a theoretical discussion on the LTC needs of the elderly and find out their influencing factors [1].

### 1.1. Current Situation of Long-Term Care in China

**Figure 1:**
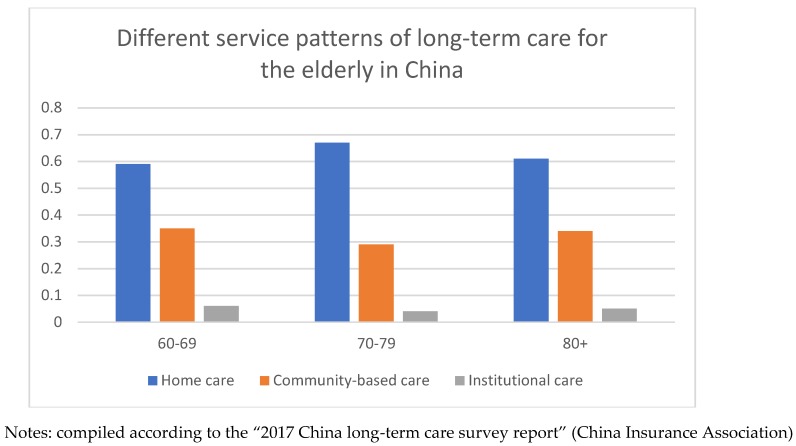
Different service patterns of long-term care for the elderly in China in 2016 (the total proportion = 1).

Informal and formal long-term care services coexist and replace each other [2]. Informal care, mainly supplied by family members, is the first choice in China with facing more and more challenges because of the changes in family structure or the information communication mode between children and the elderly. In the meantime, formal care can be provided in the form of community-based care or institutional care [3,4]. In many studies, informal and formal care were often divided into three categories—namely, home care, community-based care, and institutional care. As shown in Figure 1, the trend of specialization of long-term care service pattern is beginning to appear in China. The proportion of institutional care is increasing and making more and more progress [5]. Although home care still dominates. In terms of institutional care, the developed countries have established relatively advanced the aged care services system, the government has also introduced comprehensive policies to monitor the quality of LTC services. Compared with the developed countries, China’s medical system security is still inadequate and only 2.72% of the elderly nursing home beds. Community-based care, still in its beginning stage in China compared with developed countries, is becoming an attractive alternative due to the weakening of traditional family healthcare. Now China is facing the dilemma of how to solve this problem of limited economic resources and meet the increasing demand for services [6]. For reducing the nursing burden of the younger generation, some polices, or regulations aimed at elderly care have been published in China, such as the pilot of Long-Term Care Insurance (LTCI) policies (2016) [7].

### 1.2. Research on Benchmark Model: Anderson Model 

To integrate the factors influencing the LTC needs of the elderly, most researchers adopted self-designed models [8,9] while others used Anderson model [10,11]. The Anderson model was originally proposed in the 1960s aimed to describe and foretell the utilization of medical services [12]. Nowadays, it has been widely used to study the actual or expected use of services by the elderly [13,14].

The early forms of the Anderson model consist of three factors: predisposing characteristics, enabling factors, and need factors [15,16,17,18]. Predisposing factors are those that predate illness but may affect behaviors related to the use of long-term care, such as gender or age. Enabling factors for promoting or inhibiting the use of services in the event of disease; such as family support, insurance, etc. Need factors highlight perceived and actual nursing needs that have a direct impact on long-term care service utilization. With the parallel development of psychosocial factors in the field of LTC service needs of the elderly, the research on the utilization and needs of health services continues to develop, and the Anderson model continues to evolve. Bradley et al. found that, although the Anderson model includes ‘faith’, there is limited attention paid to the social psychological factors: “these factors for chronic and acute care maybe even more important, because the LTC aid and conventional personal tasks involved, who may have specific knowledge and strong attitude“ [19], it conforms to the program’s theory of social psychology behavior. Bradley et al. focused on expected, rather than the actual, use of an extended version of Anderson model. Considering the importance of psychosocial factors in a given cultural context, psychosocial factors were included and tested in the study of factors, influencing the expected use of care services by the elderly and caregivers [20].

In the latest version of Anderson model, three aspects were included in the psychosocial factors: (1) social patterns; (2) attitudes and awareness about the performance of LTC services; and (3) the ability of people to perceive influences on their long-term care choices [21]. Referring to some earlier studies on psychosocial factors in LTC service needs research, intergenerational relationships, unmet needs for nursing services, self-image evaluation (Table 1) corresponding to social patterns, attitudes, and knowledge were chosen as the psychosocial factors of the elderly [22].

The purpose of this study is to explore the factors that affect LTC needs of the elderly in the frame of the latest Anderson model, which added psychosocial factors to predisposing characteristics, enabling factors, and need factors to the old version. In addition, as shown in Figure 1, the choice of different long-term care service patterns is related to different age groups. So, we did three more sub-analyses—namely, by age, gender, and educational level.

## 2. Materials and Methods 

### 2.1. Data Source

Since there are few contents involving LTC in the research database such as China Health and Retirement Longitudinal Study (CHARLS) [37], we conducted our own case research in four regions—namely, Chengdu, Chongqing, Guizhou, and Hubei province—which are the main representative regions in the middle and upper reaches of the Yangtze river.

We adopted telephone survey, WeChat and QQ group survey to investigate the needs of LTC among the elderly in Chengdu, Chongqing, Guizhou, and Hubei province. The survey, conducted by the Chongqing Technology and Business University from March 2018 to December 2018, consisted of about 15 questions (please note that some variables were not included in the empirical analysis due to too many missing values) that took interviewees about 10–20 minutes to complete, and 1787 samples were recovered, of which 1308 were valid and over with a response rate of 73.1% excluding missing values. Online or verbal consent was sought from interviewees before the survey and no need for ethical approval. All investigations are conducted anonymously to respect privacy.

The sampling process consists of three steps as follows: (a) setting up a sampling framework for each region’s administrative district; (b) any district was subdivided into census districts and listing all the elderly residents in each affected district; and (c) the investigators recruited the elderly in each block using a random sample method. 

Multiple logistic regression was selected to examine the relationship between underlying factors and LTC service needs of the elderly. The latest version of Anderson’s model was incorporated into the logistics model by adding predisposing characteristics, enabling factors, need factors, and psychosocial factors. In the control of the first three groups of variables, the hierarchical model was adopted to analyze the susceptibility, initiative, and demand of each factor. Besides, the needs for LTC was stratified by gender, age, and education with multiple logistic regression analysis to further explore the differences of influencing factors among different groups (male and female, young and old).

### 2.2. Measurement Method

At the beginning of the questionnaire, we first briefly explained the basic concept of LTC to the interviewees (long term care is continuous care over a long period of time for people with chronic illness, such as cognitive impairment, or impairment, known as functional impairment), and then start asking questions.

Our paper measured LTC needs in older adults with a simple question: "which LTC way do you want to choose?" Alternative answers were: 1 (home care), 2 (community-based care), and 3 (institutional care). Based on the Anderson model (the latest version), the study evaluated four independent variables sets: predisposing characteristics, enabling factors, need factors, and psychosocial factors. Many implementation processes and methods refer to the previous research literature, such as Fu et al. [38] and Xu et al. [37].

#### 2.2.1. Predisposing Characteristics

These included age (Below/above age 69, question: How old are you?), gender (female/male, question: What’s your gender?), education level (Below Bachelor’s degree/Bachelor’s degree or above, question: What is your highest learning experience?), as well as marital status (married or not, question: What is your marital status?). In order to master the differences between different areas, the study also set "regions" (1 = Chongqing, 2 = Guizhou, 3 = Hubei, 4 = Chengdu). Unlike Fu. et al. [38] divided education level into primary school or below, junior high school or senior high school, and College or above, we divided it into below Bachelor’s degree and Bachelor’s degree or above considering the higher and fast improved education in China in the last 40 years.

#### 2.2.2. Enabling Factors

It is included: income level (question: Which of the following is your annual income?), quantity of children (question: How many children do you have?), and frequency of connection with children (question: How often do you contact the child?). Individual income or quantity of children was measured by continuous variable method.

#### 2.2.3. Need Factors

The need factor consists of two variables: IADL (Instrumental Activity of Daily Living) and quantity of chronic diseases. some sub-items of IADL were evaluated by daily life activity scale. The quantity of illnesses was calculated by question as follows: "how many chronic diseases do you have?" Higher scores represented interviewees who had more illnesses especially chronic diseases.

#### 2.2.4. Psychosocial Factors

Psychosocial factors include variables as follows: intergenerational ties, unmet needs for LTC, and self-image evaluation. (a) Intergenerational ties were measured by the following question: "How are you getting along with your children?"(answer: 1 = very poor, to, 5 = very good). The higher the score, the closer the intergenerational relationship. (b) Unmet needs for LTC were evaluated from four aspects: living surroundings, medical treatment and spiritual life. The question” Do you need the following care services” is multiple choice (0 = none,1 = only one, 2 = two, 3 = All of them). (c) The assessment of aelf-image referred to Bai et al. [39], who came up with the Chinese version of the Self-Image of Aging Scale. A higher score meant a more positive self-image.

## 3. Results

### 3.1. Statistical Characteristics Analysis

Table 2 below presents the features of the survey sample and the differences between the elderly who chose home care, community-based care, and institutional care. It is found that, among the 1308 interviewees, 75.3% (985), 16.6% (218), and 8.0% (105) chose home care, community care, and institutional care, respectively.

### 3.2. Multiple Logistic Analysis

Table 3 presents the relative risk ratio of Multiple Logistic Regression model for LTC demand, divided into induced predisposing characteristics, enabling factors, need factors, and psychosocial factors. Take community-based care as an example, it is more possible to choose home care when more confident self-image evaluation (OR = 1.0783, *p* = 0.0220).

As shown in Table 3, the elderly aged 70 or above preferred home care (OR = 1.1287) to community-based care. Marital status is an important factor, the preference for institutional care among the elderly with currently not married is particularly strong (2.4801), which is very consistent with the reality. Surprisingly, the effect of income on long-term care pattern is very small (1.0227; 0.9996). In terms of need factors, people with more chronic diseases more prefer institutional care (1.0899). In terms of self-image evaluation, the elderly with higher self-image evaluation have a lower preference for community-based care, which may be related to the fact that Chinese elderly people pay more attention to ‘personal face’ (personal dignity) and do not want to show their shortcomings to others, especially those around them.

An interesting phenomenon is that the differences between the four cases regions are very obvious. For example, compared with Chengdu, Chongqing has the highest preference for institutional care (1.3030), while Guizhou has the highest preference for home care (1.2782). These may be related to the level of economic development in different regions and the degree of market opening to the outside world.

Table 3 also reveals the model changes among the four regression models. According to the demand of LTC, four kinds of multiple logistic regression models are established. The change in model fitting is calculated. Inducers have the greatest explanatory power for LTC demand differences (pseudo-r2 = 0.0861). The addition of enabler increased the interpretation capacity by 2.7% (chi-square = 98.6076). After adding the demand factor, the improvement was 1.2% (chi-square = 110.039). The joining of psychosocial factors improved by 2.7% (chi-square = 136.5735).

### 3.3. Sub-Analysis by Gender, by Age, and by Educational Level

To discover the gender differences already exists in the factors influencing LTC needs, more models were used in this study, and the key regression of LTC needs was carried out.

There are some differences between male and female independent variables in Table 4. Regional differences, number of children or Self-image evaluation are the three remarkably correlated factors of family care preference for male, while regional differences and unsatisfied nursing service demand are the two significantly correlated factors of family care preference for female. Region, frequency of contact with children, Self-image evaluation and intergenerational relationship are the potential factors influencing the preference for the male interviewees, while marital status, regional differences and unmet care service needs are the important factors for female interviewees.

In term of age differences in the influence factors of LTC demand, the results of interviewees aged 60–69 (young) and above 70 group (older) shown in Table 5. Education level, regional differences, and unmet medical service needs are the factors most significantly connected with preference for home care in the young group, while the number of children and self-image are the most important factors in the older group. 

As shown in Table 6, from the view of predisposing characteristics, the elderly with Bachelor’s degree or above prefer institutional care to community-based care or home care. In term of regions, Chengdu, Chongqing, Guizhou, and Hubei have similar performance, but Chongqing shows the highest preference for institutional care and Guizhou was the least obvious, which may be related to the level of regional economic development or the degree of opening to the outside world. Finally, psychosocial factors, especially unmet care service needs (institutional care vs. community-based care, 1.9558) and self-image evaluation (institutional care vs. community-based care, 1.1796), have a prominent effect on the choice of the elderly with Bachelor’s degree or above compared to the elderly with below Bachelor’s degree (1.0848; 1.5245).

## 4. Discussion

This study used the latest version of Anderson’s model to explore the LTC needs of the elderly in China. We added psychosocial factors to predisposing characteristics, enabling factors, and need factors in the old version. From the sample selected from the main representative regions—namely, Chengdu, Chongqing, Guizhou, and Hubei province—in the middle and upper reaches of the Yangtze river, there were some important findings.

Firstly, predisposing characteristics play a very important role. Many factors were analyzed, for example, we support the effect of marital status as many other studies [40]. Marital status is an important factor, the preference for institutional care among the elderly who are currently unmarried is particularly strong (2.4801), which is very consistent with the reality. The discovery of regional differences may be affected by the diversity in the geographical situation, economic development, and cultural background of the four sampled regions. For example, compared with Chengdu, Chongqing has the highest preference for institutional care (1.3030), while Guizhou has the highest preference for home care (1.2782). These may be related to the level of economic development in different regions and the degree of market opening to the outside world.

Secondly, in terms of enabling factors, the research results are consistent with other research results [41,42]. The elderly people who are close to their children are more likely to “age in place” regardless of whether they receive family care or community-based care. As shown in Table 3, when them contact infrequency with children, the proportional relationship respectively was 0.7150 (home care vs. community-based care) and 2.5756 (institutional care vs. community-based care), but when they have some contact with children, the proportional relationship will be increased to 1.4532 (home care vs. community-based care). In fact, even community-based care services need to be complemented by viable care provided by family members. Because the unity between the elderly and their children is a key factor to enable the elderly to continue to live or ‘age in place’ in a familiar living environment. Surprisingly, the effect of income on long-term care pattern is very small (1.0227; 0.9996).

Thirdly, in terms of need factors, people with more chronic diseases more prefer institutional care (1.0899), which contradicted some research results [43]. It may be due to the restricted number of IADL and the disease in the sample, indicating that most interviewees were in good health. The elderly who choose community-based care were more likely to show unmet needs for care than those who choose home and institutional care. On the one hand, the limitation of community nursing sources limits the fulfillment of the elderly with community nursing sources and LTC needs unmet. On the other hand, the elderly who choose community-based care are more possible to show the needs of community-based care than those who choose home care. 

Fourthly, the role of psychosocial factors in elderly LTC service needs was abundantly demonstrated in this paper: Following to control the other three groups of factors, the variance of 2.835% can be explained by psychosocial factors. This confirms the significant role of psychosocial factors in influencing LTC needs, consistent with previous research results [15]. Meaningful correlations between psychosocial factors and LTC service needs indicate something. Table 3 also reveals the model changes among the four regression models. According to the demand of LTC, four kinds of multiple logistic regression models are established. The change in model fitting is calculated. Inducers have the greatest explanatory power for LTC demand differences (pseudo-r2 = 0.0861). The addition of enabler increased the interpretation capacity by 2.7% (chi-square = 98.6076). After adding the demand factor, the improvement was 1.2% (chi-square = 110.039). The joining of psychosocial factors improved by 2.7% (chi-square = 136.5735). In terms of self-image evaluation, the elderly with the higher self-image evaluation has the lower preference for community-based care, which may be related to the fact that Chinese elderly people pay more attention to ‘personal face’ (personal dignity) and do not want to show their shortcomings to others, especially those around them. Self-image evaluation is composed of several factors such as general physical health, attitude towards life or psychosocial states [39]. People who have positive self-perceptions about their physical health, attitudes to life, and social status recognition mostly think they have the ability of taking good care of themselves, are more active to involve social interactions, and therefore willing to accept home care. In traditional Chinese cultural values, the elderly attach great importance to the views of the society or others on them. They will try their best to show better aspects to the outside world, and whether they can be taken care of by their families is one of the important aspects. 

At the end of the empirical analysis, regression analysis by gender/age/educational level group was conducted in order to further examine the differences in factors affecting LTC service needs in the elderly. (a) From the perspective of gender, it is a difference in the mainly influencing factors of LTC service needs for men or women among the elderly. As shown in Table 4, taking marital status as an example, the difference between male and female is significant when them currently not married (OR = 1.4080 and 4.1832 in institutional care vs. community-based care). The role of men or women in social construction and the gender division in labor market may help to clarify the LTC needs of elderly people affected by gender differences. (b) In terms of age, the influencing factors for the two-age group (age in 60–69 and above 70) are completely different. This may be because in China, the life expectancy is about 75, the elderly over 69 years may detect the urgency of the demand for LTC while the elderly under 69 years think this demand is more distant. (c) From the view of educational level, psychosocial factors—especially unmet care service needs and self-image evaluation—played an important role in the choice of the elderly. Unmet care service needs (institutional care vs. community-based care, 1.9558) and self-image evaluation (institutional care vs. community-based care, 1.1796), have a prominent effect on the choice of the elderly with Bachelor’s degree or above compared to the elderly with below Bachelor’s degree (1.0848; 1.5245).

## 5. Conclusions

The growing trend of an aging population stressed the urgency of the task in the fund collection of long-term care (LTC) services for the disabled elderly. This research, though based on findings in the Chinese context, could afford a reference value for other countries, especially those that similarly emphasize familial relationships.

This study aims to explore the factors that affect LTC needs of the elderly in the frame of the latest Anderson Model, which added psychosocial factors to predisposing characteristics, enabling factors, and need factors in the old version. In fact, few researches studying the role of psychosocial factors in influencing factors of LTC needs of the elderly and testing educational-related differences in this subject in China. The sample is selected the main representative regions—namely, Chengdu, Chongqing, Guizhou, and Hubei province—in the middle and upper reaches of the Yangtze river. It found some interesting phenomena, but the results may be slightly inaccurate due to the selection of regions and the design of questionnaires, which needs to be further improved and deepened in the future research. In addition, we did three more sub-analyses—namely, by age, by gender, and by educational level. They are useful supplement, but perhaps more factors, such as health insurance purchase status or long-term care insurance (LTCI) purchase intention, can be taken into account in future studies.

## Figures and Tables

**Table 1:** Research on the psychosocial factors associated with long-term care needs

Factors	Research Literature	Conclusion
Intergenerational relationship	Chou (2010) and Wang (2016) [23,24]	Intergenerational relationship had a significant impact on the LTC needs of the elderly
	Tian & Wang (2014) [25] and Komter & Vollebergh (2002) [26]	Intergenerational relationship had no significant impact on the LTC needs of the elderly
Unmet needs	Choi & McDougall (2009) and Xu & Chen (2019) [27,28]	Unmet needs may negatively affect older adults or their providers, such as increased risk and insecurity of health problems or signs of depression
	Tennstedt, McKinlay, & Kasten (1994) [29]	Understanding the unmet needs of care services of the elderly can be used as an indicator of future needs of care services
Self-image evaluation	Lai (2009) [30], Zhang. et al. (2006) [31], Barak. et al. (2001) [32], Boduroglu. et al. (2006) [33]	Self-image evaluation of the elderly is rapidly declining
	Chiu &Y u (2001) [34] and Chow & Bai (2011) [35]	The traditional image of the elderly is now being questioned
	Wei & Li (2013) [36]	Negative self-image evaluation that makes the elderly tend to restrain themselves and not express their needs

**Table 2:** Features of interviewees choosing home care, community-based care, or institutional care.

Factors	Total (N = 1308)		Home Care (N = 985)		Community-Based Care (N = 218)		Institutional Care (N = 105)		*p*
	N	%	N	%	N	%	N	%	
**Predisposing characteristics**									
Age									0.198
60–69	673	51.45	492	49.95	122	55.96	59	56.19	
70 or above	635	48.55	493	50.05	96	44.04	46	43.81	
Gender									0.206
Female	622	47.55	453	45.99	115	52.75	53	50.48	
(Male)	686	52.45	532	54.01	103	47.25	52	49.52	
Educational level									0.018
Below Bachelor’s degree	954	72.94	737	74.82	151	69.27	66	62.86	
(Bachelor’s degree or above)	354	27.06	248	25.18	67	30.73	39	37.14	
Marital status									0.177
Currently not married	224	17.13	163	16.55	36	16.51	26	24.76	
(Currently married)	1084	82.87	822	83.45	182	83.49	79	75.24	
Region									0.001
CQ(Chongqing)	497	38.00	358	36.35	83	38.07	57	54.29	
GZ(Guizhou)	278	21.25	198	20.10	74	33.94	6	5.71	
HB(Hubei)	283	21.64	216	21.93	38	17.44	29	27.62	
(CD)(Chengdu)	250	19.11	213	21.62	23	10.55	13	12.38	
**Enabling factors**									
Income (thousand, RMB) (mean, SD)	37.62, 25.12		37.40, 25.94		38.62, 24.11		37.48, 18.67	0.040	
Number of children (mean, SD)	2.33, 1.23		2.41, 1.24		2.10, 1.19		2.05, 1.16	0.001	
Contact frequency with children								<0.001	
Infrequently	47	3.59	25	2.54	9	4.13	12	11.43	
Sometimes	150	11.47	109	11.07	23	10.55	18	17.14	
(Frequently)	1111	84.94	851	86.39	186	85.32	75	71.43	
**Need factors**									
IADL (mean, SD)	7.69, 1.66		7.72, 1.61	7.59, 1.75			7.59, 1.71	0.455	
Number of chronic diseases (mean, SD)	1.23, 1.01		1.19, 1.04	1.36, 1.08			1.39, 1.05	0.032	
**Psychosocial factors**									
Intergenerational relationships (mean, SD)	4.94, 0.89		4.99, 0.80	4.65, 0.88			4.36, 1.16	<0.001	
Unmet care service needs (mean, SD)	4.81, 3.76		4.67, 3.57	5.42, 3.69			4.05, 3.22	0.001	
Self-image evaluation (mean, SD)	57.32, 9.02		57.66, 8.45	53.01, 8.46			54.88, 9.77	0.019	

**Table 3:** Multiple logistic regression results for long-term care service needs

Factors	Home Care vs. Community-Based Care		Institutional Care vs. Community-Based Care	
	*p*	OR	*p*	OR
**Predisposing characteristics**				
Age				
70 or above	0.7602	1.1287	0.8011	0.9534
(60–69)				
Gender				
Female	0.1270	0.7991	1.0447	1.0521
(Male)				
Educational level				
Below Bachelor’s degree	0.2167	1.8923	1.0256	1.0548
(Bachelor’s degree or above)				
Marital status				
Currently not married	0.8032	1.1277	0.0168	2.4801
(Currently married)				
Region				
CQ	0.0094	0.4977	0.6573	1.3030
GZ	0.0000	1.2782	0.0011	0.1449
HB	0.0052	0.4011	0.9649	0.9964
(CD)				
**Enabling factors**				
Income	0.4809	1.0227	0.5029	0.9996
Number of children	0.0115	1.3314	0.6594	1.1308
Contact frequency with children				
Infrequently	0.4252	0.7150	0.1207	2.5756
Sometimes	0.2751	1.4532	0.0609	2.2890
(Frequently)				
**Need factors**				
IADL	0.3843	1.1077	0.3990	0.9681
Number of chronic diseases	0.1617	0.9271	0.8232	1.0899
**Psychosocial factors**				
Intergenerational relationships	0.7161	1.0983	0.0199	0.7098
Unmet care service needs	0.0094	0.9828	0.0283	0.9576
Self-image evaluation	0.0220	1.0783	0.0273	1.0962
Changes in model fits for long-term care needs (*** *p* < 0.001)				
Regression models		Pseudo R2	change	Chi-square
Predisposing		0.0861		73.4034 ***
Predisposing and enabling		0.1144	0.0283	98.6076 ***
Predisposing, enabling, and need		0.1270	0.0126	110.039 ***
Predisposing, enabling, need, and psychosocial		0.1554	0.0283	136.5735 ***

**Table 4:** Multiple logistic regression results for long-term care needs by gender

Factors	Male				Female			
	Home Care vs. Community-Based Care		Institutional Care vs. Community-Based Care		Home Care vs. Community-Based Care		Institutional Care vs. Community-Based Care	
	*p*	OR	*p*	OR	*p*	OR	*p*	OR
**Predisposing characteristics**								
Age								
70 or above	0.3549	1.3891	0.7906	1.2169	0.4924	0.8505	0.4011	0.7003
(60–69)								
Educational level								
Below Bachelor’s degree	0.3997	1.5649	0.5047	1.2355	0.3787	1.8864	0.3412	0.5973
(Bachelor’s degree or above)								
Marital status								
Currently not married	0.315	0.7129	0.6489	1.4080	0.1197	1.7493	0.0042	4.1832
(Currently married)								
Region								
CQ	0.0430	0.3927	0.9461	0.9607	0.1218	0.5848	0.4578	1.6716
GZ	0.0011	0.2058	0.0378	0.1522	0.0042	0.3402	0.0157	0.1155
HB	0.0168	0.2919	0.9177	0.9250	0.1522	0.5134	1.0206	1.0773
(CD)								
**Enabling factors**								
Income	0.2016	0.9964	1.0332	1.0510	0.5512	1.0951	0.3003	0.9166
Number of children	0.0094	1.5372	0.2761	1.3555	0.2194	1.2285	0.7644	0.9723
Contact frequency with children								
Infrequently	1.0405	1.0584	0.0199	7.0759	0.3969	0.6079	0.3223	0.29295
Sometimes	0.7654	1.2054	0.357	1.869	0.1806	1.9246	0.0745	3.0544
(Frequently)								
**Need factors**								
IADL	0.7675	1.0153	0.1522	0.8589	0.1543	1.1781	0.7833	1.09725
Number of chronic diseases	0.5313	0.9607	0.6457	1.1676	0.3129	0.9261	0.7675	1.1214
**Psychosocial factors**								
Intergenerational relationships	0.5617	1.16235	0.01995	0.59745	1.0248	1.0458	0.3591	0.8295
Unmet care service needs	0.3591	1.0122	0.8379	1.0332	0.0042	0.9492	0.0136	0.91035
Self-image evaluation	0.0304	1.0909	0.0199	1.1235	0.3423	1.0668	0.4788	1.071

**Table 5:** Multiple logistic regression results for long-term care needs by age

Factors	60–69				70 or above			
	Home Care vs. Community-Based Care		Institutional Care vs. Community-Based Care		Home Care vs. Community-Based Care		Institutional Care vs. Community-Based Care	
	*p*	OR	*p*	OR	*p*	OR	*p*	OR
**Predisposing characteristics**								
Gender								
Female	0.8232	0.9838	0.6972	1.2432	0.0556	0.6205	0.7801	0.9051
(Male)								
Educational level								
Below Bachelor’s degree	0.0278	2.4132	0.5977	1.0756	0.7324	0.9018	0.6899	0.8232
(Bachelor’s degree or above)								
Marital status								
Currently not married	0.22155	1.7598	0.00525	5.21325	0.7497	0.9366	0.29505	1.7934
(Currently married)								
Region								
CQ	0.0105	0.2415	0.3549	0.5061	0.1858	0.6541	0.273	2.0485
GZ	0.0000	0.1092	0.0052	0.0945	0.0136	0.3958	--	--
HB	0.0021	0.1585	0.0861	0.2436	0.6195	0.8064	0.0672	4.2892
(CD)								
**Enabling factors**								
Income	0.7014	1.0279	0.3717	0.9471	0.6667	1.0206	0.7885	1.0857
Number of children	0.1638	1.3041	0.4882	0.8599	0.0073	1.4731	0.2079	1.3555
Contact frequency with children								
Infrequently	0.0535	0.3496	0.9051	0.9219	0.4389	2.5956	0.0252	17.5087
Sometimes	0.8589	0.9649	0.6835	0.8032	0.1134	2.4108	0.0021	8.0860
(Frequently)								
**Need factors**								
IADL	0.4977	0.9397	1.0101	1.0363	0.6982	1.0825	0.5029	0.9712
Number of chronic diseases	0.0997	0.8368	0.1743	1.4038	0.42	0.9471	0.3895	0.8673
**Psychosocial factors**								
Intergenerational relationships	0.8526	1.0878	0.0252	0.63	0.9555	1.0699	0.4315	0.8263
Unmet care service needs	0.0283	0.9670	0.0126	0.9009	0.147	0.9964	0.6079	1.0143
Self-image evaluation	0.462	1.0626	0.0336	1.1119	0.0294	1.092	0.4284	1.0762

**Table 6:** Multiple logistic regression results for long-term care needs by educational level

Factors	Below Bachelor’s Degree				Bachelor’s Degree or Above			
	Home Care vs. Community-Based Care		Institutional Care vs. Community-Based Care		Home Care vs. Community-Based Care		Institutional Care vs. Community-Based Care	
	*p*	OR	*p*	OR	*p*	OR	*p*	OR
**Predisposing characteristics**								
Age								
70 or above	0.0726	1.4586	0.8301	1.2777	0.5170	0.8930	0.4211	0.7353
(60–69)								
Gender								
Female	0.0644	1.4464	0.5865	0.6879	0.1036	2.1520	0.1940	0.4178
(Male)								
Marital status								
Currently not married	0.0307	0.7485	0.6813	1.4784	0.1256	1.8367	0.0044	1.3923
(Currently married)								
Region								
CQ	0.0452	0.9123	0.9933	1.2087	0.1278	0.9140	0.4806	1.3551
GZ	0.0011	0.7160	0.0396	0.7598	0.0044	0.7572	0.0165	0.8212
HB	0.0176	0.8064	0.9635	0.9113	0.1598	0.8391	1.0716	0.9311
(CD)								
**Enabling factors**								
Income	0.2116	1.0462	1.0848	1.1036	0.5788	1.1499	0.3153	0.9624
Number of children	0.0099	1.6140	0.2899	1.4233	0.2304	1.2899	0.8026	1.0209
Contact frequency with children								
Infrequently	1.0925	1.1113	0.0209	7.4297	0.4167	0.6383	0.3384	0.3075
Sometimes	0.8037	1.2656	0.3748	1.9624	0.1896	2.0208	0.0782	3.2071
(Frequently)								
**Need factors**								
IADL	0.3059	1.0661	0.1598	0.9018	0.1620	1.2370	0.8224	1.0521
Number of chronic diseases	0.1578	1.0087	0.6780	1.2259	0.3285	0.9724	0.8059	1.1774
**Psychosocial factors**								
Intergenerational relationships	0.4898	1.2204	0.0209	0.6273	1.0760	1.0980	0.3770	0.8709
Unmet care service needs	0.3770	1.0628	0.8797	1.0848	0.0044	0.9966	0.0143	1.9558
Self-image evaluation	0.0319	1.1454	0.0209	1.1796	0.3594	1.1201	0.5027	1.5245
